# Methods for Collecting and Analyzing Post-Ejaculatory Uterine Fluid and the Uterus in Mice

**DOI:** 10.21769/BioProtoc.5544

**Published:** 2025-12-20

**Authors:** Yu Matsumoto, Ban Sato, Masafumi Inui, Manato Sunamoto, Natsuko Kawano, Kenji Miyado

**Affiliations:** 1 Laboratory of Regulatory Biology, Department of Life Sciences, School of Agriculture, Meiji University, Kanagawa, Japan; 2 Laboratory of Animal Regeneration Systemology, Department of Life Sciences, School of Agriculture, Meiji University, Kawasaki, Japan; 3 Department of Reproductive Biology, National Research Institute for Child Health and Development, Tokyo, Japan; 4 Division of Diversity Research, National Research Institute for Child Health and Development, Tokyo, Japan

**Keywords:** Mating, Internal fertilization, Intrauterine environment, Biochemical analysis, Histological analysis, Reproductive biology

## Abstract

In mammals, the semen is ejaculated into the female reproductive tract, and the sperm travel to the oviduct to fertilize the egg. A comprehensive understanding of the pre- and post-ejaculatory intrauterine environment is one of the key points for overcoming infertility; however, the dynamics of the intrauterine environment and its physiological role in the uterus, namely in the internal fertilization process, remain unclear. Conventional methods for collecting uterine fluids from the uterus post-ejaculation of mice show challenges regarding the ambiguous ejaculation timing. Here, we established a method for a mating environment with exact ejaculation timing. We also created a simple method for collecting pre- and post-ejaculatory uterine fluid without using forceps. Our methods achieved time-dependent biochemical and histological analyses of uterine fluids to provide fundamental information regarding protein composition and uterine structure changes during pre- and post-ejaculation. This protocol is suitable for analyzing temporal changes in reproductive phenomena, thereby contributing to elucidating the physiological role of the uterus in the process of intrauterine fertilization.

Key features

• This protocol is used for the simple collection of pre- and post-ejaculatory uterine fluid.

• Changes in the pre- and post-ejaculatory intrauterine environment can be examined by controlling the dissection time of females after ejaculation.

• An estrous female can be determined without a vaginal smear test in this protocol.

• This protocol can be used to analyze the protein composition of post-ejaculatory uterine fluid and is applicable to analyze sperm within the uterus post-ejaculation.

## Graphical overview


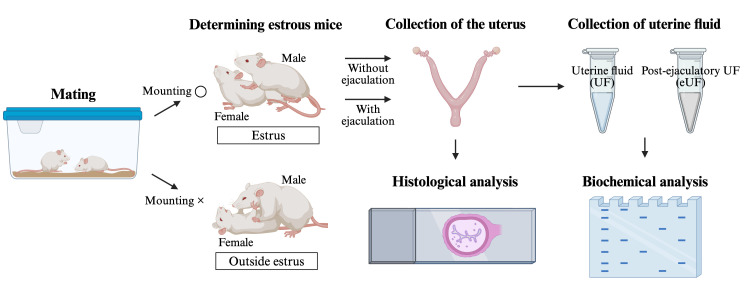


**Methods for collecting and analyzing post-ejaculatory uterine fluid and the uterus**

## Background

The female reproductive tract comprises the vagina, uterus, oviducts, and ovaries. Mammals reproduce by internal fertilization, whereby the male ejaculates semen into the uterus or vagina. Ejaculated sperm fertilize eggs through a complex process in the female reproductive tract [1]. Mice, which share mechanistic features with humans in reproductive phenomena such as internal fertilization and implantation, are extremely useful as model animals. Previous studies in mice have reported that regulation of sperm migration in the uterotubal junction (UTJ) and sperm storage and capacitation in the oviduct are essential subprocesses of fertilization [2–4]. Although a recently published article suggested that an abnormal fluid environment within the post-ejaculatory uterus contributes to infertility [5], the role of the post-ejaculatory uterus in internal fertilization remains unclear compared to the UTJ and oviduct. To address this issue, it is necessary to establish protocols that allow evaluation of pre-ejaculatory, post-ejaculatory (immediately post-ejaculation or pre-fertilization), and post-ejaculatory (post-fertilization) intrauterine environment. The conventional protocol for collecting post-ejaculatory uterine fluid (eUF) from the uterus post-ejaculation is limited in that it cannot collect eUF immediately after post-ejaculation. Therefore, we propose a simple method for collecting pre-and post-ejaculatory (immediately post-ejaculation) uterine fluid and uterus, followed by biochemical and histological analyses. This protocol provides a novel approach to study dynamics of the uterus, sperm (motility, capacitation, and survival), and fertilization in internal fertilization, which is expected to contribute to elucidating the physiological role of the uterus.

## Materials and reagents

**Biological materials**

1. ICR male and female mice (Japan SLC, Inc.)

**Reagents**

1. Ethanol 70% (Yoshida Pharmaceutical Company, Ecosyoueta Disinfectant Solution, catalog number: 14987288980046)

2. Pierce^TM^ bovine serum albumin standard ampules, 2 mg/mL (Thermo Fisher Scientific, catalog number: 23209)

3. Pierce^TM^ BCA Protein Assay kit (FUJIFILM Wako Pure Chemical Corp., catalog number: 297-73101)

4. Nunc^TM^ MicroWell^TM^ 96-well, Nunclon Delta-treated, flat-bottom microplate (Thermo Fisher Scientific, catalog number: 167008)

5. 2-Mercaptoethanol (Sigma-Aldrich, catalog number: M314835406-91)

6. Sodium dodecyl sulfate (SDS) (FUJIFILM Wako Pure Chemical, catalog number: 191-07145)

7. Glycerol (FUJIFILM Wako Pure Chemical, catalog number: 075-00616)

8. Bromophenol blue (FUJIFILM Wako Pure Chemical, catalog number: 029-02912)

9. Tris(hydroxymethyl)aminomethane (Tris) (NACALAI TESQUE, Inc., catalog number: 35406-91)

10. Hydrochloric acid (HCl) (FUJIFILM Wako Pure Chemical, catalog number: 080-01066)

11. Acrylamide/bis solution 37.5:1 at 30% (Bio-Rad Laboratories, Inc., catalog number: 1610158)

12. N,N,N',N'-Tetramethylethylenediamine (TEMED) (FUJIFILM Wako Pure Chemical, catalog number: 205-06313)

13. Ammonium persulfate (APS) (FUJIFILM Wako Pure Chemical, catalog number: 802811)

14. Urea (FUJIFILM Wako Pure Chemical, catalog number: 219-00175)

15. Glycine (FUJIFILM Wako Pure Chemical, catalog number: 077-00735)

16. Protein molecular weight marker (FUJIFILM Wako Pure Chemical, catalog number: 234-02464)

17. Coomassie brilliant blue R-250 (CBB-R250) (Thermo Fisher Scientific, catalog number: 20278)

18. Acetic acid (FUJIFILM Wako Pure Chemical, catalog number: 017-00251)

19. Methanol (FUJIFILM Wako Pure Chemical, catalog number: 131-01826)

20. 10× Phosphate-buffered saline (PBS) (TOHO Co., Ltd., catalog number: 12-9423-5)

21. Bouin's solution (FUJIFILM Wako Pure Chemical, catalog number: 023-17361)

22. Sucrose (FUJIFILM Wako Pure Chemical, catalog number: 196-00015)

23. Tissue-Tek^®^ Cryomold^®^ (Sakura Finetek Japan Co., Ltd., catalog number: 4557)

24. Optimal cutting temperature (OCT) compound (Sakura Finetek Japan, catalog number: 45833)

25. Liquid nitrogen

26. New hematoxylin solution type M (Muto Pure Chemicals, catalog number: 30142)

27. Eosin Y solution at 1% (Muto Pure Chemicals, catalog number: 32002)

28. Ethanol 100% (Muto Pure Chemicals, catalog number: 43105)

29. Xylene (Muto Pure Chemicals., catalog number: 43122)

30. Mounting medium (New M·X) (Matsunami Glass Industry Co., Ltd., catalog number: FX00500)

**Solutions**

1. Tris/HCl (pH 6.8, pH 8.8), 1 M (see Recipes)

2. 6× Sample buffer (see Recipes)

3. SDS 10% (see Recipes)

4. APS 25% (see Recipes)

5. Stacking gel solution (see Recipes)

6. Separation gel solution (see Recipes)

7. Running buffer (see Recipes)

8. CBB staining solution (see Recipes)

9. CBB decolorizing solution (see Recipes)

10. Eosin solution (see Recipes)

**Recipes**

**1. Tris/HCl (pH 6.8, pH 8.8), 1 M**

**Table:** 

Reagent	Final concentration	Quantity or volume
Tris	1 M	60.57 g
Adjust to pH 6.8 and pH 8.8 with HCl		
Distilled water	n/a	<500 mL
Total	n/a	500 mL

*Note: pH 6.8 and pH 8.8 Tris/HCl solutions are prepared separately.*

**2. 6× sample buffer**

**Table:** 

Reagent	Final concentration	Quantity or volume
Tris/HCl (pH 6.8), 1 M	350 mM	3.5 mL
2-Mercaptoethanol	30% (v/v)	3 mL
SDS	10% (w/v)	1 g
Glycerol	30% (v/v)	3 mL
Bromophenol blue	0.06% (w/v)	6 mg
Distilled water	n/a	0.5 mL
Total	n/a	10 mL

**3. SDS 10% (w/v)**

**Table:** 

Reagent	Final concentration	Quantity or volume
SDS	10% (w/v)	20 g
Distilled water	n/a	<200 mL
Total	n/a	200 mL

**4. APS 25% (w/v)**

**Table:** 

Reagent	Final concentration	Quantity or volume
APS	25% (w/v)	2.5 g
Distilled water	n/a	<10 mL
Total	n/a	10 mL

**5. Stacking gel solution**

**Table:** 

Reagent	Quantity or volume
30% Acrylamide/bis solution 37.5:1	375 μL
Tris/HCl (pH 6.8), 1 M	468.75 μL
SDS, 10% (w/v)	37.5 μL
TEMED	3 μL
Distilled water	2856.25 μL
Total	3740.6 μL

**6. Separation gel solution**

**Table:** 

Reagent	Quantity or volume
30% Acrylamide/bis solution 37.5:1	2000 μL
Tris/HCl (pH 8.8), 1 M	2812.5 μL
SDS, 10% (w/v)	75 μL
TEMED	6 μL
Distilled water	2587.5 μL
Total	7481 μL

**7. Running buffer**

**Table:** 

Reagent	Final concentration	Quantity or volume
Tris	25 mM	3.03 g
SDS	0.1% (w/v)	1 g
Glycine	192 mM	14.4 g
Distilled water	n/a	<1,000 mL
Total	n/a	1,000 mL

**8. CBB staining solution**

**Table:** 

Reagent	Final concentration	Quantity or volume
CBB R-250	0.25% (w/v)	0.5 g
Acetic acid	10% (v/v)	20 mL
Methanol	50% (v/v)	100 mL
Distilled water	n/a	80 mL
Total	n/a	200 mL

**9. CBB decolorizing solution**

**Table:** 

Reagent	Final concentration	Quantity or volume
Acetic acid	7.5% (v/v)	15 mL
Methanol	25% (v/v)	50 mL
Distilled water	n/a	135 mL
Total	n/a	200 mL

**10. Eosin solution**

**Table:** 

Reagent	Quantity or Volume
1% Eosin Y solution	100 mL
60% ethanol	500 mL
Acetic acid	0.6 mL
Total	600.6 mL

**Laboratory supplies**

1. Disposable latex gloves (ASKUL, catalog number: WE59721)

2. Microcentrifuge tubes 1.5 mL (Greiner Bio-One Co., Ltd., catalog number: 616201)

3. Centrifuge tubes 15 mL (AS ONE, catalog number: 4-3632-01)

4. Pipette tips 10 μL (WATSON, catalog number: 110-207C)

5. Pipette tips 200 μL (WATSON, catalog number: 110-705C)

6. Pipette tips 1,000 μL (WATSON, catalog number: 110-7-6C)

7. Gel loading tips 200 μL (BM Equipment Co., Ltd., catalog number: 010-Q)

8. Kimwipes (NIPPON PEPAR CRECIA Co., Ltd., catalog number: 62020)

9. Paper towels (ASKUL, catalog number: 1944368)

10. Dish 60 mm in size (AGC TECHNO GLASS Co., Ltd., catalog number: 1010-060)

11. Absorbent paper (ATTO Corp., catalog number: CB-06A)

12. Glass slide (Muto Pure Chemicals Co., Ltd., catalog number: 513617)

13. 24 mm × 50 mm cover glass (thickness: 0.13–0.17 mm) (Matsunami Glass Industry, catalog number: C024501)

## Equipment

1. Personal protective equipment (e.g., mask, goggles, and lab coats)

2. Precision balance (Mettler Toledo, model: PB602-S)

3. P-20 pipette (Gilson, model: F123600)

4. P-200 pipette (Gilson, model: F123601)

5. P-1000 pipette (Gilson, model: F120602)

6. Small straight scissors (Natsume Seisakusho, model: B-12)

7. Large straight scissors (Natsume Seisakusho, model: B-3)

8. Tweezers (AS ONE, model: 2-529-12)

9. Plastic cages (Clea Japan, Inc., model: CL-0103-2 Mouse TPX)

10. Water bottles, rubber stoppers (Clea Japan, Inc., model CL-0904)

11. SpectraMax (Molecular Devices, model: SpectraMax^®^ iD5e)

12. 1 mm dual mini gel cast (glass plates, seal gasket, comb, and clips) (ATTO, model: AE-6401)

13. pH meter (HIRIBA, model: F-72)

14. Beaker glass, 500 mL (AS ONE, model: 2-5091-06)

15. Magnetic stirrer (Thermo Fisher Scientific, model: Magnetic stirrer RT Basic-12)

16. Stirrer (AS ONE, model: 3-6657-02)

17. Electrophoresis system (ATTO, model: WSE-1100)

18. Rocking mixer (AS ONE, model: 1-5829-22)

19. Cryostat (Thermo Fisher Scientific, model: CryoStar NX50)

20. Microscope (Keyence Corp., model: BZ-X700)

## Software and datasets

1. Microsoft Excel version 16.102.1 (Microsoft Corporation)

2. BioRender (https://www.biorender.com/)

## Procedure

**A. Animals**

**Figure 1.**
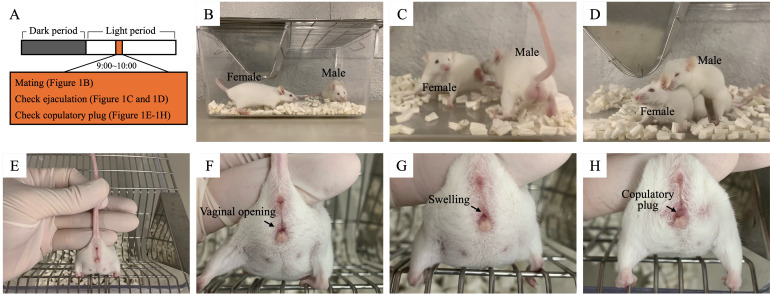
Mating male and female mice. (A) Experimental flow. (B) Male and female mice housed together. (C) Refusal of mounting behavior. (D) Allowance of mounting behavior. (E–H) Copulatory plugs checked. (F) Vagina of a female (during proestrus, metestrus, or diestrus) that refused mounting behavior. (G) Vagina of a female (during estrus) that allowed mounting behavior. The arrow indicates swelling of the vulva. (H) Vagina of a female post-ejaculation.

1. Breed ICR strain male and female mice separately (1–5 mice per cage) under the following specific pathogen-free conditions: controlled temperature (23 ± 1 °C), humidity (40%–60%), light/dark cycles (lights on at 5 am and off at 7 pm), and ad libitum access to food and water (Figure 1A).

**B. Mating, determining estrous mice, and assessing copulatory plugs**

1. House male and female mice (8 weeks old) together between 9 and 10 am (Figure 1B).

*Note: Ad libitum access to food and water is suspended during this time.*

2. Check mounting behavior on the female (Figure 1C and D).

*Note: If there is no mounting behavior about 10 min after housing mice, change male or female. Outside estrous females refuse mounting behavior (Video 1), whereas estrous females allow it (Video 2).*

**Video 1.**
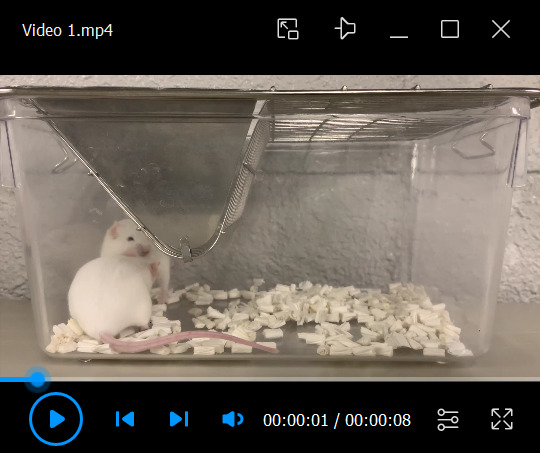
Mating male and female outside estrus

**Video 2.**
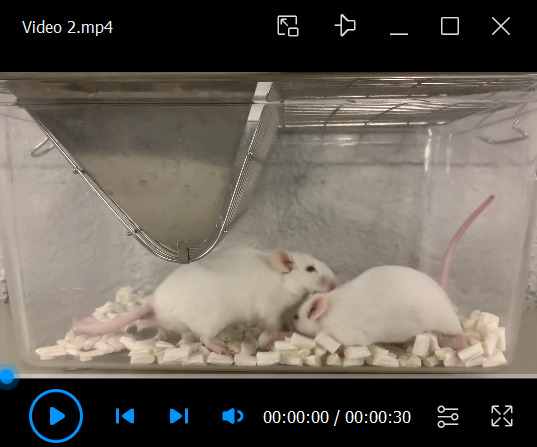
Mating male and female in estrus

3. Check ejaculation into the female.

*Note: Ejaculation can be confirmed by the male’s temporary rigidity while holding the female after mounting behavior with anteroposterior movement of the loin (Video 1).*

4. Lift the base of the female’s tail and check for a copulatory plug attached to the vagina using tweezers (Figure 1E–H).

*Note: If no mounting behavior is observed about 10 min after housing mice, change male or female. In females post-ejaculation, a copulatory plug can be observed blocking the vaginal opening. This plug formation occurs within minutes after ejaculation is confirmed.*

**C. Collection of eUF**

**Figure 2.**
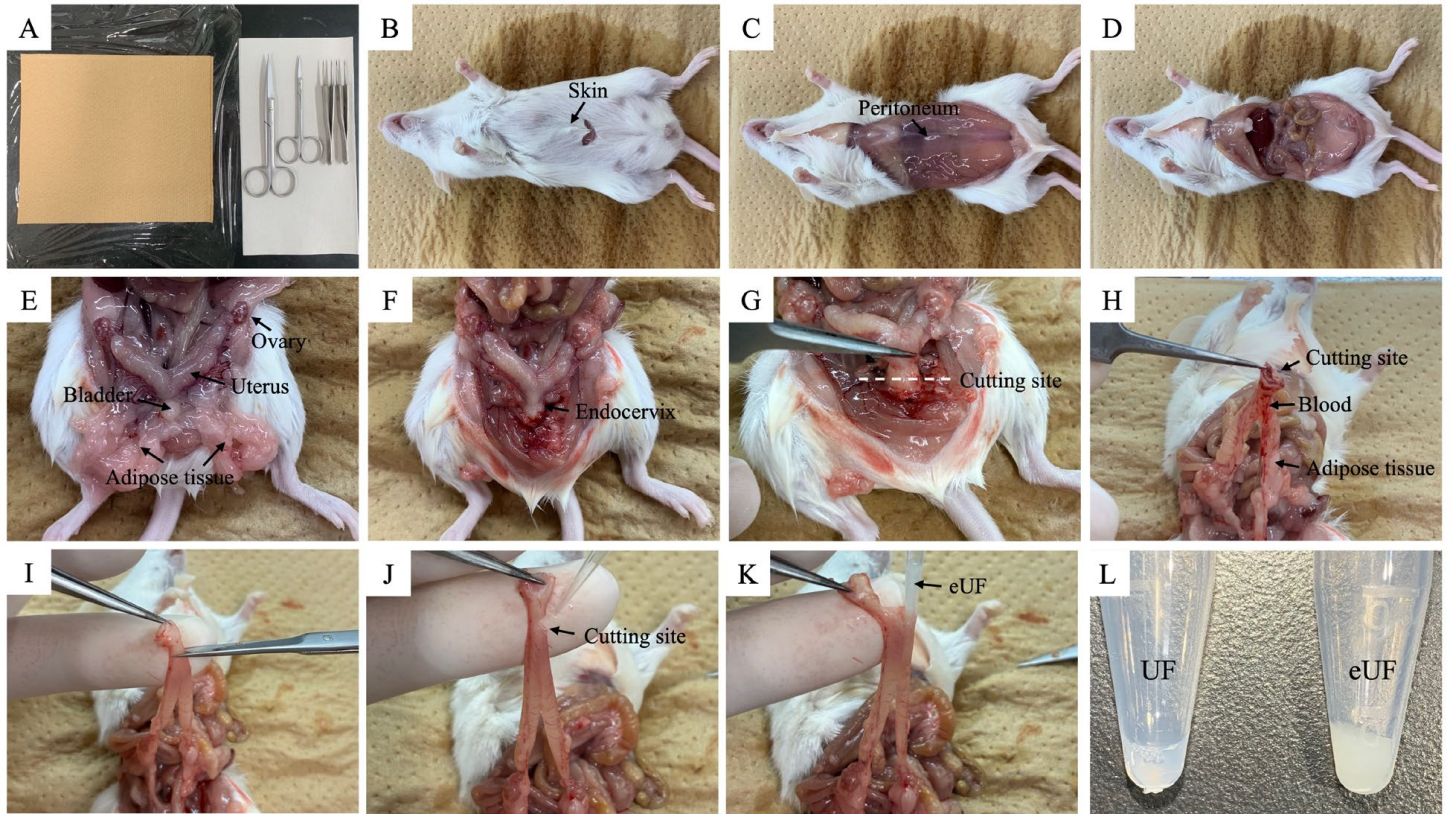
Collection of post-ejaculatory uterine fluid (eUF). (A) Tools for abdominal exploration. (B) Sacrificed female mice post-ejaculation. (C) Peritoneum accessed. (D) Cut peritoneum. (E) The location of the female reproductive tract. (F) Removal of the bladder and adipose tissue. (G, H) Cut endocervix. (I) A small part of the uterus is cut open. (J) A P-200 pipette with a 200 μL tip is inserted into the uterus. (K) Collection of eUF from the uterus. (L) UF and eUF collected from the uterus.

1. Place scissors and tweezers on the bench and wear gloves (Figure 2A).

2. Sacrifice females post-ejaculation.

*Note: As a method of euthanasia, hold the base of the tail with one hand, place the thumb and index finger of the other hand on the neck, and quickly pull both sides (head and tail side) to perform cervical dislocation. For the exact time of ejaculation, refer to step B3.*

3. Disinfect the abdomen with 70% ethanol and cut the skin using large scissors (Figure 2B).

4. Pull apart the two sides (head and tail side) of the cut skin to access the peritoneum (Figure 2C).

5. Cut the peritoneum using small scissors (Figure 2D) and find the female reproductive tract (V-shaped uterus, oviducts, and ovaries) using tweezers (Figure 2E).

6. Remove the bladder and adipose tissue around the bladder using small scissors and tweezers (Figure 2F).

7. Cut the endocervix using small scissors (Figure 2G) and hold it using tweezers (Figure 2H).

8. Remove adipose tissue and blood from the uterus using tweezers and Kimwipes and excise a portion of the uterus (Figure 2I).

9. Collect eUF in a 1.5 mL tube using a P-200 pipette (Figures 2J–L).

*Note: By following steps C1–C9, pre-ejaculatory uterine fluid (UF) can be collected from estrus females (steps B1 and B2). Section C (collection of eUF) can be performed in approximately 5 min.*

**D. Preparation of protein concentration for SDS-PAGE**

1. Prepare UF and eUF diluted 10× and 30× with distilled water.

*Note: When the viscosity of eUF is high, it is extremely difficult to measure the appropriate amount with a pipette. In such cases, add distilled water to the eUF to decrease its viscosity before preparing the sample.*

2. Prepare a dilution series of 1, 0.5, 0.25, 0.125, 0.0625, and 0.03125 mg/mL albumin using bovine serum albumin standard ampules (2 mg/mL).

3. Prepare samples and a dilution series in a 96-well plate using the Protein Assay BCA kit.

4. Measure the absorbance of samples and a dilution series using the SpectraMax iD5.

5. Prepare a calibration curve with absorbance on the x-axis and protein concentration on the y-axis, and a calibration curve of the dilution series using Excel.

6. Identify the protein concentration of UF and eUF using the calibration curve as an indicator.

7. Adjust the protein concentrations of UF and eUF (1 μg/μL and 1× sample buffer) using 6× sample buffer and distilled water.

8. Store samples at -80 °C until use.

**E. Preparation of acrylamide gel (8%) for SDS-PAGE**

1. Set the seal gasket on the glass plates and assemble the gel caster using clips.

2. Insert the comb into the gel caster and place a mark 5 mm below the comb.

3. Wear gloves and prepare stacking and separation gel solutions in 15 mL tubes.

4. Add 25% (w/v) APS (25 μL) to the separation gel solution.

5. Apply the separation gel solution containing APS up to the mark into the gel caster.

*Note: After adding 25% APS to the separation gel solution, mix well and promptly add the solution to the gel caster.*

6. Slowly add 1 mL of distilled water using a P-1000 pipette into the gel caster.

7.When the gel hardens, tilt the gel caster and remove the distilled water using Kimwipes.

8. Add 25% (w/v) APS (12.5 μL) to stacking gel solution.

9. Apply stacking gel solution containing APS into the gel caster and insert a comb.

*Note: After adding APS to the stacking gel solution, mix well and promptly add the solution to the gel caster.*

10. When the gel hardens, cover it with plastic wrap and store at 4 °C until use.

**F. Comparison of the protein composition between UF and eUF using SDS-PAGE**

1. Add 8 M urea in distilled water (5 μL) to samples (12.5 μL; step D6) and incubate at 95 °C for 5 min.

2. After incubation, add 6× sample buffer (1 μL).

4. Remove the comb and clips from the gel caster and place the gel in the electrophoresis system.

5. Fill the electrophoresis system with running buffer.

6. Wash the inside of the wells using a P-200 pipette with a 200 μL tip.

7. Apply the samples (18.5 μL) and protein molecular weight marker (18.5 μL; protein molecular weight marker: 10 μL; 1× sample buffer: 2.5 μL; 6× sample buffer: 1 μL; 8 M urea in distilled water: 5 μL) in the wells using a P-20 pipette with a gel loading tip.

8. Apply 1× sample buffer containing urea in the empty wells (18.5 μL; 1× sample buffer: 12.5 μL; 6× sample buffer: 1 μL; 8M urea in distilled water: 5 μL).

9. Allow the gel to electrophorese (select mode: Tris-Gly/PAGEL, 1 gel, 80 min).

10. After electrophoresis, place the gel in a case containing CBB staining solution and stain for 1 h at room temperature (25–27 °C) on a shaking apparatus.

11. Wash the gel with tap water and decolorize with CBB decolorizing solution.

**Figure 3.**
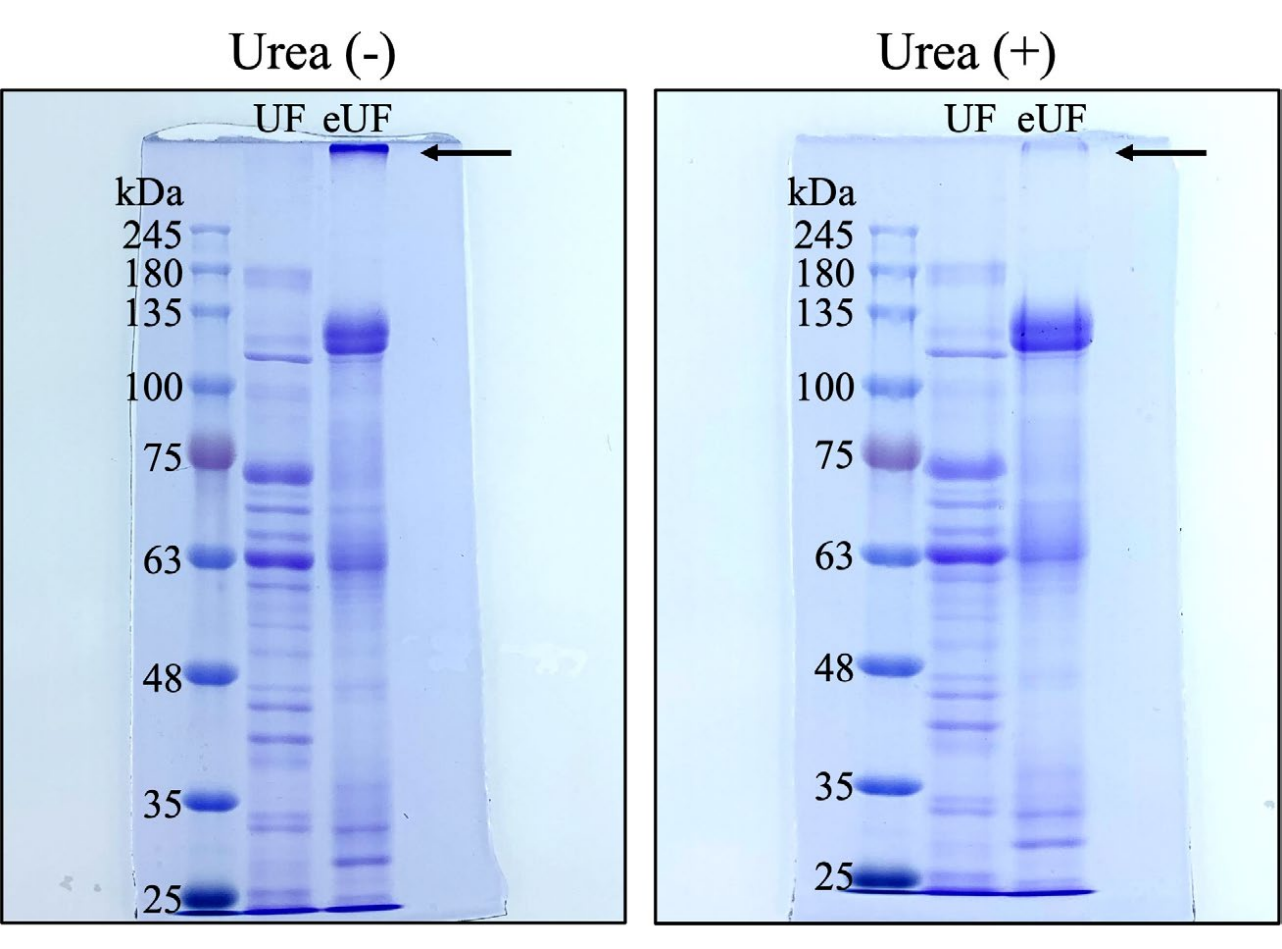
SDS-PAGE of post-ejaculatory uterine fluid (eUF). Results of SDS-PAGE of UF and eUF samples with urea (right image) or without urea (left image). Arrow: clumping proteins.

*Note: eUF proteins separate more efficiently upon the addition of urea (Figure 3).*

**G. Histological analysis of the uterus post-ejaculation using hematoxylin-eosin (H&E) staining**

**Figure 4.**
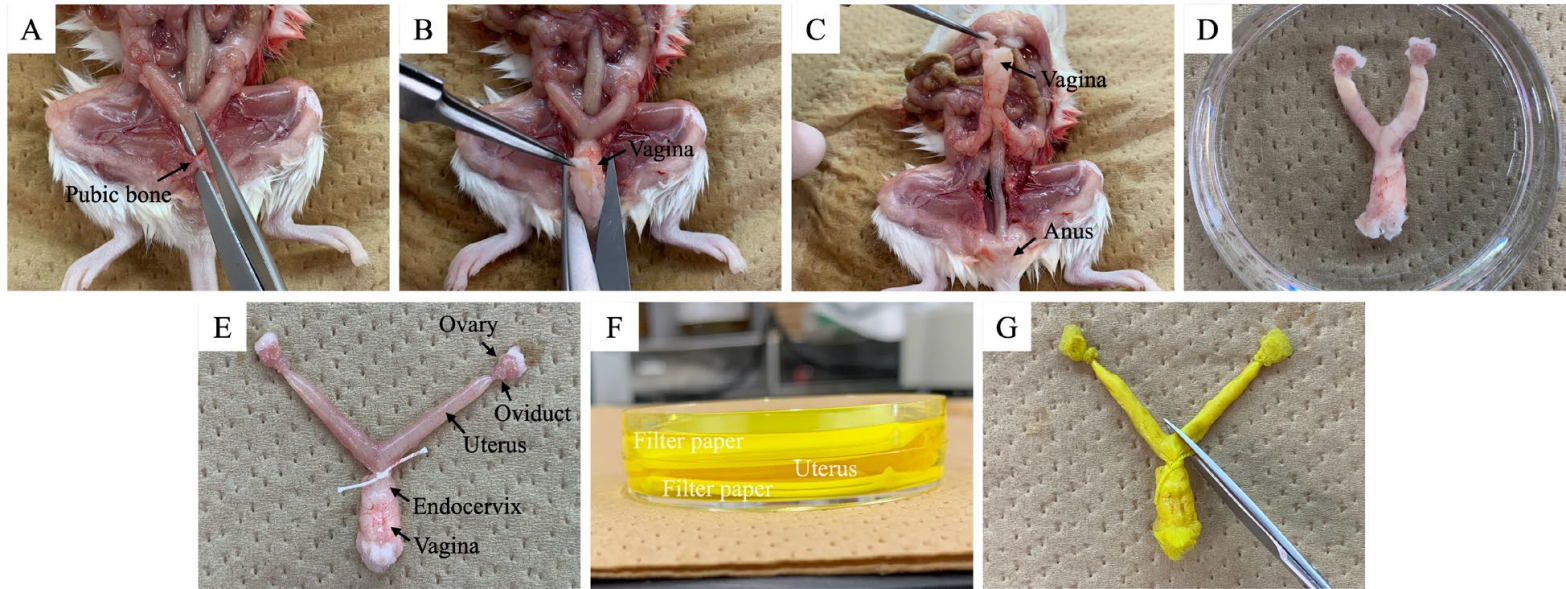
Collection of the uterus post-ejaculation. (A) Cut pubic bone. (B) Cut between the vagina and anus. (C, D) The uterus collected post-ejaculation. (E) Ligation of the upper side of the vaginal cervix. (F) Uterus placed between filter papers and fixed. (G) Cutting of uterus.

1. Prepare females post-ejaculation (steps B1–3) as well as the dissection environment (step C1).

2. Sacrifice females (steps C2) and locate the female reproductive tract (steps C3–6).

3. Cut the pubic bone and then cut between the vagina and anus using large scissors (Figure 4B).

4. Lift the vagina with tweezers (Figure 4C) and use small scissors to collect the female reproductive tract, including the vagina, into a 60 mm dish (Figure 4D).

5. Use tweezers to ligate the upper side of the vaginal cervix (uterine side) with silk thread.

6. Place the female reproductive tract between filter papers and fix with Bouin's solution overnight at 4 °C.

7. Wash the female reproductive tract with 1× PBS for 10 min at room temperature and replace with 30% (w/v) sucrose solution overnight at 4 °C.

8. Cut the uterus using small scissors and embed in a cryomold using OCT compound.

9. Freeze the samples in liquid nitrogen and store at -80 °C until use.

10. Prepare 5 μm sections using the cryostat and attach to glass slides.

11. Dry the sections thoroughly at room temperature, then wash with tap water for 10 min.

12. Stain the sections with hematoxylin solution for 5 min, wash with tap water, and stain with an eosin solution for 2 min.

13. Wash the sections with tap water and sequentially immerse in 70%, 80%, 90%, 100%, and 100% ethanol, xylene, and xylene for 30 s each.

14. Add a few drops of New M·X to the sample, and cover with a cover glass.

15. Capture brightfield images of the uterus using a microscope (Figure 5).

**Figure 5.**
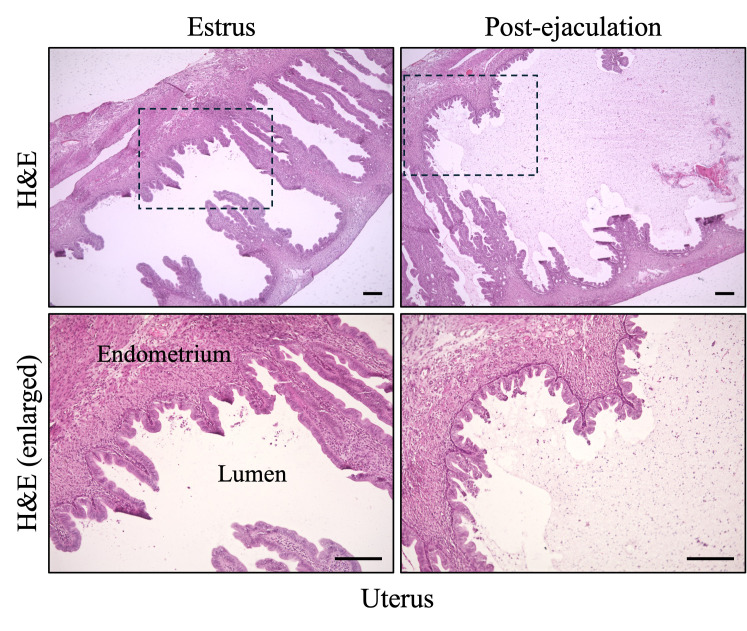
Histological analysis of the uterus post-ejaculation. Histological analysis of the uterus during estrus and post-ejaculation using H&E staining. Dotted box, image enlarged below; scale bars = 100 μm.

## Validation of protocol

Part of this protocol is an optimized version of the method used in previous articles:

- Kawano et al. [6]. Seminal vesicle protein SVS2 is required for sperm survival in the uterus. *Proc Natl Acad Sci USA*. (SI Appendix, Figures S9–S13)
- Matsumoto et al. [7]. Dynamics of post-ejaculated intrauterine environment in mice. *MicroPubl Biol*. (Figure 1)

## General notes and troubleshooting

**General notes**

1. In conventional protocols, estrous females used for mating are determined based on cytological analysis obtained from a daily vaginal smear test. This protocol, which determines estrous mice based on mounting behavior during mating, is more efficient than conventional protocols from an experimental handling perspective (step B2). This method of determining estrous mice can be applied to analyses such as egg collection and artificial insemination in estrous mice.

2. This protocol has demonstrated reproducibility not only in ICR but also in C57BL/6N strain mice (data not shown). However, further investigation is required regarding its applicability to other rodents.

**Troubleshooting**

**Problem 1:** No mounting behavior is observed during mating between male and female mice (step B2).

Possible cause: Age of mice used for mating.

Solution: Immature or aged mice (especially females) may not exhibit mounting behavior and ejaculation. Pay attention to these mice and perform mating.
